# Supported Gold Catalysts for Base-Free Furfural Oxidation: The State of the Art and Machine-Learning-Enabled Optimization

**DOI:** 10.3390/ma16196357

**Published:** 2023-09-22

**Authors:** Joëlle Thuriot-Roukos, Camila Palombo Ferraz, Hisham K. Al Rawas, Svetlana Heyte, Sébastien Paul, Ivaldo Itabaiana Jr, Mariusz Pietrowski, Michal Zieliński, Mohammed N. Ghazzal, Franck Dumeignil, Robert Wojcieszak

**Affiliations:** 1 Université de Lille, CNRS, Centrale Lille, Université d’Artois, UMR 8181-UCCS-Unité de Catalyse et Chimie du Solide, 59000 Lille, France; joelle.thuriot@univ-lille.fr (J.T.-R.); hisham.alrawas@univ-lille.fr (H.K.A.R.); svetlana.heyte@univ-lille.fr (S.H.); sebastien.paul@centralelille.fr (S.P.); franck.dumeignil@univ-lille.fr (F.D.); 2 Department of Inorganic Chemistry, Institute of Chemistry, Federal University of Rio de Janeiro, Rio de Janeiro 221941-910, Brazil; cpferraz@iq.ufrj.br; 3 Department of Biochemical Engineering, School of Chemistry, Federal University of Rio de Janeiro, Rio de Janeiro 21941-910, Brazil; ivaldo@eq.ufrj.br; 4 Faculty of Chemistry, Adam Mickiewicz University, 61-614 Poznań, Poland; mariop@amu.edu.pl (M.P.); mardok@amu.edu.pl (M.Z.); 5 Institut de Chimie Physique (ICP), UMR 8000 CNRS, Université Paris-Saclay, 91400 Orsay, France; mohammed-nawfal.ghazzal@paris-saclay.fr

**Keywords:** decision tree, machine learning, furfural, oxidation, gold, base-free

## Abstract

Supported gold nanoparticles have proven to be highly effective catalysts for the base-free oxidation of furfural, a compound derived from biomass. Their small size enables a high surface-area-to-volume ratio, providing abundant active sites for the reaction to take place. These gold nanoparticles serve as catalysts by providing surfaces for furfural molecules to adsorb onto and facilitating electron transfer between the substrate and the oxidizing agent. The role of the support in this reaction has been widely studied, and gold–support interactions have been found to be beneficial. However, the exact mechanism of furfural oxidation under base-free conditions remains an active area of research and is not yet fully understood. In this review, we delve into the essential factors that influence the selectivity of furfural oxidation. We present an optimization process that highlights the significant role of machine learning in identifying the best catalyst for this reaction. The principal objective of this study is to provide a comprehensive review of research conducted over the past five years concerning the catalytic oxidation of furfural under base-free conditions. By conducting tree decision making on experimental data from recent articles, a total of 93 gold-based catalysts are compared. The relative variable importance chart analysis reveals that the support preparation method and the pH of the solution are the most crucial factors determining the yield of furoic acid in this oxidation process.

## 1. Introduction

In 2022, approximately 82% of global energy consumption still relied on fossil resources [1,2,3]. However, the limited nature of these resources and the consequent release of CO_2_ into the atmosphere have led to concerns about climate change and global warming [4,5]. As a result, there has been a growing interest in non-edible biomass, such as lignocellulose, as a renewable alternative [6]. Lignocellulose is abundant, sustainable, and cost-effective, making it an attractive source for organic chemicals and liquid biofuels [7,8]. Moreover, its production consumes CO_2_, which makes it more environmentally friendly compared to fossil resources [5]. Lignocellulose primarily consists of cellulose (40–50%), hemicellulose (25–35%), and lignin (15–20%) [7,9,10], making it a promising feedstock for biorefineries [11,12,13,14,15,16].

**Scheme 1:**
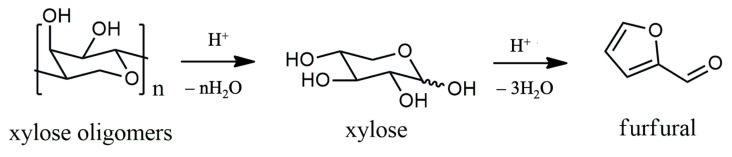
Production route of furfural from hemicellulose.

The conversion of biomass to biofuels can be achieved through various methods such as pyrolysis, liquefaction, or gasification under harsh conditions [17,18]. However, a more attractive and sustainable approach is the hydrolysis of cellulose and hemicellulose, providing access to second-generation biofuels and platform molecules [2,5,7,19,20,21,22,23,24,25,26,27]. These platform molecules include monosaccharides (glucose and xylose), polyols (glycerol, arabitol, and sorbitol), furfurals (furfural and *5*-hydroxymethylfurfural), and acids (*2,5*-furandicarboxylic acid, levulinic acid, and lactic acid) [7,12,13,20]. Among these, furfural (FFR) stands out as a crucial link between raw biomass resources and biofuels and chemicals. Furfural is typically obtained by dehydrating xylose, a hemicellulosic pentose present in agricultural wastes like corn stalks (as shown in Scheme 1). The global production of furfural is estimated at 280,000 tons/year, with 90% of it produced in China, the Dominican Republic, and South Africa [28,29].

Despite the early establishment of a commercial facility for furfural production in 1921, current industrial-scale production remains low at approximately 50%. This is primarily due to undesirable side reactions, including decomposition into smaller oxidized compounds and condensation between furfural and humic reaction intermediates or other furfural molecules. Furfural’s chemical structure, featuring a heteroaromatic furan ring and an aldehyde group, enables its transformation into valuable chemicals. The aldehyde group can undergo oxidation to form an acid or directly participate in oxidative esterification. Over the past two decades, numerous catalyst systems have been investigated for the oxidative conversion of furfural. These systems include noble-metal-based catalysts, non-noble-metal-based catalysts, and combinations of both. Noble metals like gold and ruthenium are known for their high intrinsic oxidation activities. The optimization of catalytic formulations and the development of new ones require extensive research taking into account various factors, such as active metals, supports, metal additives, co-catalysts, and catalyst preparation methods. Significant progress has been made in catalytic performances by creating multi-phase formulations (bimetallic, promoted monometallic) and refining active phase preparation methods that directly impact nanoparticle size and distribution [7,10,11,12]. In this work, we specifically focused on the effect of catalyst composition on selectivity in liquid-phase furfural oxidation under uncontrolled pH conditions (base-free conditions). We focused also on the significant role of furfural in a biorefinery and its potential for conversion into valuable chemical intermediates through various catalytic pathways. The main objective of this study was to comprehensively review the research conducted in the last five years on the catalytic oxidation of furfural under base-free conditions. To the best of our knowledge, no review article has systematically analyzed the specific catalyst formulations and their performances in this transformation process. By establishing a deeper understanding of the relationship between catalyst active sites and oxygen-involving reactions, we aim at facilitating more efficient developments in this field. Additionally, we conducted a machine learning analysis of the published articles and using a tree decision to study the available catalytic data.

## 2. Catalytic Oxidation of Furfural

**Scheme 2:**
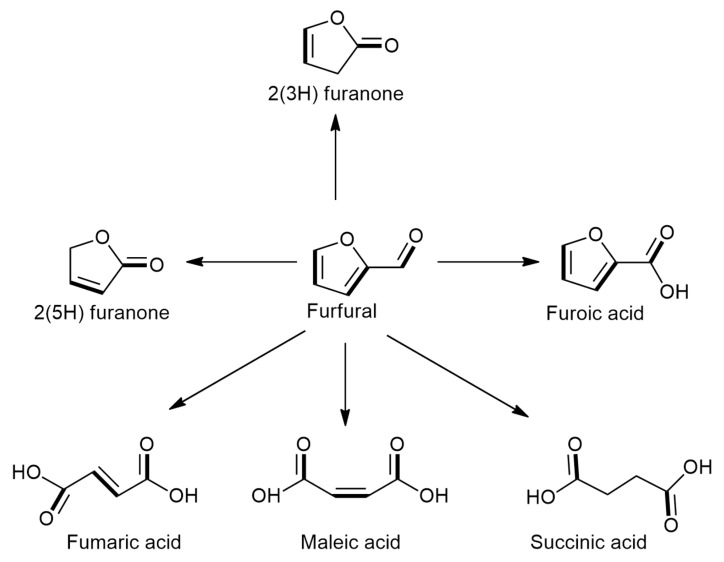
Main products that could be obtained via oxidation of furfural.

The oxidation of furfural can yield intriguing products like furoic acid, maleic acid, and succinic acid (Scheme 2).

Gold-based catalysts have demonstrated significant potential in aerobic oxidation of various substrates in water, prompting extensive research on their application in furfural oxidation within basic solutions. However, the synthesis of furoic and maleic acids from furfural under uncontrolled pH conditions (without the addition of a base) [30,31] using gold catalysts presents challenges and lacks comprehensive documentation. Until now, studies have primarily focused on the oxidation of low-molecular-weight sugars under strongly alkaline conditions, with the majority of the literature centered around controlled, alkaline pH conditions [32,33]. Under high-pH conditions, glucose undergoes complete conversion into gluconic acid. However, several studies indicate that at elevated pH levels, glucose becomes chemically unstable, and it decomposes. Notably, in the presence of K_2_CO_3_, thermal oxidative decomposition of glucose was observed at 70 °C after 4 h of reaction, without the aid of a catalyst. Similar findings have been documented in the existing literature [34,35,36], confirming the generation of diverse carboxylic acids. Moreover, in alkaline pH environments, substrate isomerization can occur, and various chemical intermediates coexist in the reaction solution. Hence, the development of efficient catalysts capable of selectively converting furfural without the need for a base becomes critically important. In the field of base-free oxidation of furfural, researchers have made significant progress, but there are still several critical gaps and challenges that warrant further investigation and research. Despite substantial research efforts, the exact mechanism of furfural oxidation under base-free conditions is not yet fully understood. A critical gap exists in uncovering the intricate details of the reaction pathways, intermediates, and the roles of different catalyst components. In addition, various catalysts have been explored for furfural oxidation (not only gold-based), and there is room for improving their efficiency and selectivity. The main gap is still the development of novel catalyst materials based on stable non-noble metals (or oxides) and a deeper understanding of structure–activity relationships. An example could be the partial dealloying of the bimetallic nanoparticles. There is no study discussing this aspect in the case of liquid-phase oxidation. As we try to show in this review, the choice of support plays a crucial role in the overall performance of the catalyst (yield to furoic acid). However, very often, the full characterization of the support is not shown (performed). Furthermore, detailed kinetic and thermodynamic studies are necessary to gain a deeper understanding of the rate-limiting steps. No articles recently published present a complete kinetic study of this reaction on gold catalysts. In summary, while there have been significant advancements in the field of base-free oxidation of furfural, there are still several critical gaps that require further research and exploration to advance our understanding and application of this important chemical transformation. Addressing these gaps will contribute to the development of more efficient and sustainable processes in biorefineries and related industries.

## 3. Furfural Oxidation on Basic Supports

As mentioned earlier, the presence of alkaline conditions has proven to significantly enhance the efficiency of gold catalysts. However, a groundbreaking patent was granted in 2017 for a novel process that enables the synthesis of furoic acid without the use of any added inorganic base, employing gold as the catalyst [37]. This invention introduced an exceptionally efficient heterogeneously catalyzed method for producing furoic acid in the liquid phase, yielding an impressive 98% furoic acid. The novelty of this invention lies in the development of highly effective heterogeneous catalysts operating in non-alkaline solutions, which, as previously discussed, is a challenging endeavor.

**Figure 1:**
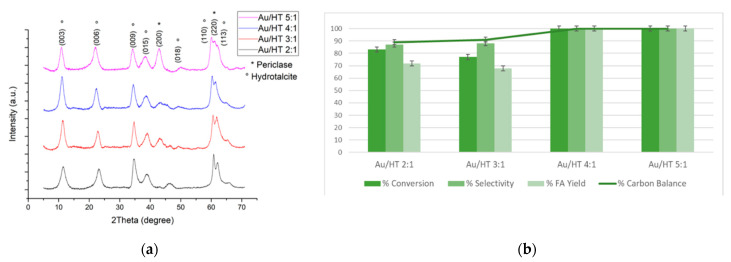
(**a**) Comparison of XRD patterns of the Au/HT catalysts, with gold nanoparticles (2 wt.%) supported on hydrotalcite carrier; (**b**) comparison of the performance in furfural oxidation of Au/HT catalysts with hydrotalcite supports taking different Mg:Al molar ratios. Batch reactor, *p* = 6 bar (O_2_), t = 110 °C, 2 h, 600 rpm, FFR/Au molar ratio = 200:1, [FFR] = 2266 mM, 25 mg of catalyst. Reprinted with permission from [38].

The most favorable results were achieved using gold nanoparticles (2 wt.%) supported on hydrotalcite carriers. The nature of the support was identified as a crucial factor, prompting further exploration of various known basic carriers for the furfural oxidation reaction. Initial investigations focused on gold nanoparticles deposited on different types of hydrotalcites [38]. Hydrotalcites are known for their basicity, which heavily depends on the Mg:Al ratio used during synthesis. Several hydrotalcite carriers were synthesized through co-precipitation at various Mg:Al molar ratios (5:1, 4:1, 3:1, and 2:1) [38]. After calcination at 500 °C for 3 h, the hydrotalcite structure transformed into MgO-Al_2_O_3_ mixed oxides. XRD studies confirmed the typical features of a Mg(Al)O-type mixed oxide, and the samples were found to be free from any impurities, including residual nitrate salts. Furthermore, XRD analyses of the gold-modified samples revealed characteristic reflections corresponding to hydrotalcite (Figure 1a). The hydrotalcite structure demonstrated its memory effect, effectively restoring its original form after the gold nanoparticle deposition process, triggered by the rehydration of the mixed oxides. The catalytic activity of all tested catalysts was observed in the oxidation of furfural under liquid-phase conditions at 110 °C and 6 bar of oxygen (Figure 1b).

As expected, the introduction of gold nanoparticles in the catalysts led to a significant increase in both furfural conversion and furoic acid yield compared to the use of supports alone [38]. Gold catalysis is known to hinder the formation of radical reaction pathways [39], resulting in lower furfural degradation, as evidenced by the higher carbon balance (consistently close to or above 90%). Furthermore, the influence of varying Mg:Al molar ratios on catalytic properties was investigated to assess the potential enhancement of basic physicochemical properties [38,40]. As illustrated in Figure 1b, an increase in furfural conversion and furoic acid yield was observed with higher Mg content in Au/HT catalysts. Remarkably, complete conversion and 100% selectivity to furoic acid were achieved with Au/HT 4:1 and Au/HT 5:1 catalysts [38]. This improvement could be attributed to the overall increase in basicity of the support, which effectively promoted catalyst activity. To monitor any possible metal leaching during the reaction, ICP-OES analysis of liquid-phase samples was performed. Encouragingly, the results revealed no detectable leaching of gold even after a 2 h reaction period.

**Figure 2:**
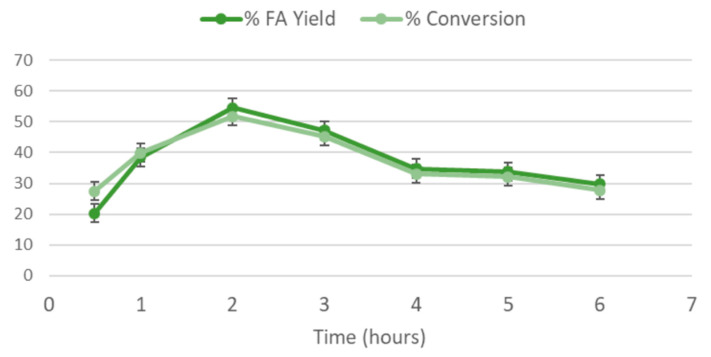
Furfural oxidation in CSTR employing Au/HT 4:1 as catalyst. P = 5 bar air; t = 110 °C; FFR/Au = 50; feed: 0.5 mL min^−1^ [FFR] = 26 mM; 100 mg of catalyst. Reprinted with permission from [38].

However, the concentration of Mg in the post-reaction mixture steadily increased with reaction time, indicating gradual leaching. In addition, the pH of all samples after the reaction was 7, which evidenced in situ formation of a base, even if Mg leaching was limited to 10 mgL^−1^ [38]. Based on the results of ICP-OES analysis, the Au/HT 4:1 catalyst was also tested in a continuous-stirred-tank reactor (CSTR) operating at a constant flow rate of the reaction mixture (0.5 mL min^−1^). The continuous reaction system allows better monitoring of the stability of the catalyst and its suitability for potential industrial applications. In this system, the reactant was pumped into the tank and the reaction solution was drawn off at the same rate, while the catalyst remained trapped inside the reactor with a filter to prevent possible entrainment. Figure 2 shows the course of the reaction over 6 h [38].

Initially, the catalyst exhibited relatively low activity, which gradually increased until the second hour of the reaction but then progressively declined. Nevertheless, across the course time of the reaction, high selectivity to furoic acid and a consistent carbon balance were maintained. Notably, a steady state was not achieved during the 6 h reaction, indicating ongoing catalyst degradation (due to the leaching discussed above), even during the initial two hours. During the reaction, the pH was continuously monitored and remained constant at 7, similar to the reaction conducted in the batch reactor. It is essential to consider that maintaining the pH at 7 required stronger leaching compared to the batch reactor setup since a fresh solution of furfural (initial pH = 3) was continuously introduced into the CSTR reactor. As a result, the leaching of the carrier occurred continuously. The proposed explanation for the declining activity lies in the gradual release and removal of homogeneous Mg(OH)_2_ during the reaction, carried away by the reactant stream [38,39,40]. This phenomenon contributed to the decrease in catalyst activity over time. On the basis of ICP analysis and pH measurements, it was shown that in situ formation of a homogeneous base occurs as a result of carrier dissolution under hydrothermal conditions. Ultimately, this work provided evidence that the use of HT carrier-based catalysts under the aforementioned hydrothermal conditions, should not be considered in terms of base-free conditions.

**Figure 3:**
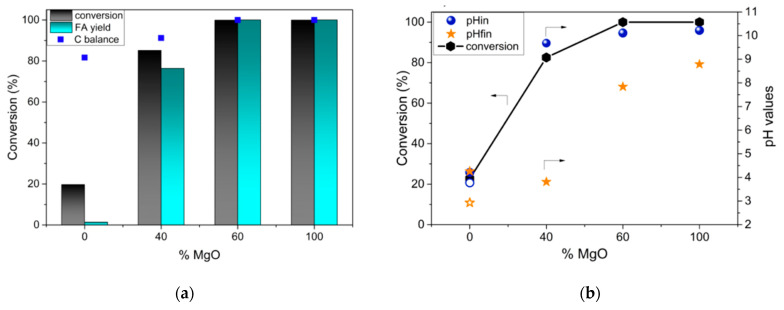
Base-free furfural oxidation using Au-based catalysts supported on MgF_2_-MgO with various relative MgF_2_ and MgO amounts. (**a**) Furfural conversion and FA yield versus % MgO; (**b**) furfural conversion and initial and final pH values versus % MgO (solid symbols) and blank test (open symbol) (49.4 μmol, substrate/metal = 50 (moL/moL), air (26 bar), 110 °C, 600 rpm, 2 h). pH*_i_*_n_ indicates the pH measured before reaction (furfural solution + catalyst) and pH*_fin_* indicates the pH measured after reaction. Reprinted with permission from [41].

Since the presence of MgO was determined to be crucial for achieving high catalyst activity, leading to in situ formation of OH- ions, further investigations were conducted using MgO to explore methods of stabilizing it within the catalyst to prevent leaching. Consequently, a new catalytic system was proposed, involving the deposition of gold on a support based on the binary material MgF_2_-MgO [41]. To obtain MgF_2_ and MgF_2_-MgO materials with different ratios of MgF_2_ to MgO, magnesium carbonate and hydroxide were reacted with corresponding amounts of a 40 wt.% aqueous hydrofluoric (HF) acid solution. The amount of HF was carefully chosen to yield final materials with 40, 60, and 100 mol% MgF_2_. However, the Au/MgF_2_ catalyst demonstrated relatively low activity, comparable to zirconium and silica catalysts. Moreover, the presence of the catalyst had no discernible effect on the pH of the reaction medium, and there was no evidence of carrier leaching either. Consequently, the degradation and/or side reactions, along with the lower catalytic performance, were attributed to the acidic nature of the reaction environment, which proved to be unfavorable for the active centers of gold-based catalysts. By reducing the MgF_2_ content within the binary support system (MgF_2_-MgO), a notable enhancement in conversion, furoic acid (FA) yield, and carbon balance was observed [39], as depicted in Figure 3a. The catalytic performance of Au/MgF_2_-MgO was remarkably effective in the oxidation of furfural to FA, achieving yields as high as 99% after just 2 h of reaction time. Remarkably, the inclusion of 60 mol% magnesium oxide in the mixed system (MgF_2_-MgO) proved sufficient to deliver the same level of activity as that of pure MgO under identical conditions. This indicates that Au/MgF_2_-MgO catalysts not only exhibit high activity but also enable pH adjustments, making them potentially suitable for industrial-scale applications [41]. To this end, pH measurements were conducted in the furfural solution (blank) and the reaction mixture, both before and after the reaction (Figure 3b).

Each catalytic solution containing the MgO phase exhibited an initial pH of approximately 10 (furfural + catalyst) (Figure 3b, pH*_in_*), indicating partial dissolution of MgO. Subsequent pH measurements after the reaction revealed an increase in leaching of the support with higher MgO content in the solid phase (Figure 3b, pH*_fin_*). However, for the catalyst containing 40 mol. % MgO, almost complete conversion of furfural was observed, resulting in more than 70% furoic acid (FA) content and a final pH of 3.8. This pH level is crucial since the acids are fully protonated at this point (*pKa* = 3.12 for FA), facilitating direct acid isolation. The ability to achieve high conversion at low pH*_in_* and simultaneously obtain high product yields at low pH*_fin_* represents a highly promising development. This promising result allows for direct FA production without the need for alkali or subsequent neutralization, which opens up the possibility of expanding its application [41].

As shown above, catalysts based on solid base supports such as HT and MgO promoted catalytic transformations in aqueous media. However, the partial dissolution of MgO in water at low pH is an important issue. For this reason, studies on other alkali metal oxides from group two—BeO, BaO, CaO, and SrO [42]—were performed. Since these oxides are also soluble in water, studies of furfural oxidation were performed in methanol, which should lead to the synthesis of furoic acid methyl ester. At the same time, the use of organic solvent should prevent metal leaching. Gupta et al. [43] conducted the oxidative esterification of furfural with propanol, leading to high yields of propyl 2-furoate using a Au/HT catalyst, O_2_, and K_2_CO_3_ as the base. Similarly, Kegnæs et al. explored the oxidative dehydrocondensation of alcohols with *N*-hexylamine to obtain *N*-hexylamides [44]. They observed that the oxidation of furfural with a Au/TiO_2_ catalyst and CH_3_O-K as a homogeneous base resulted in high yields of a methyl ester. However, this process required a significant amount of alkali, and it would be more environmentally friendly and cost-effective if no alkali were used. Another proposed approach involved the oxidative esterification of furfural with methanol without the addition of any base [45]. In this case, gold supported on sulfated zirconium oxide exhibited superior catalytic properties compared to the Au/TiO_2_ catalyst. The authors suggested that the improved activity of these materials was due to the presence of very small Au particles, enabling efficient O_2_ dissociation. This process generates atomic oxygen with strong alkaline properties, facilitating the easy activation of CH_3_OH and enhancing the reaction rate [45]. Despite the significant increase in the use of gold catalysts in oxidation and oxidative esterification, the challenge of base utilization remains unresolved [42].

**Figure 4:**
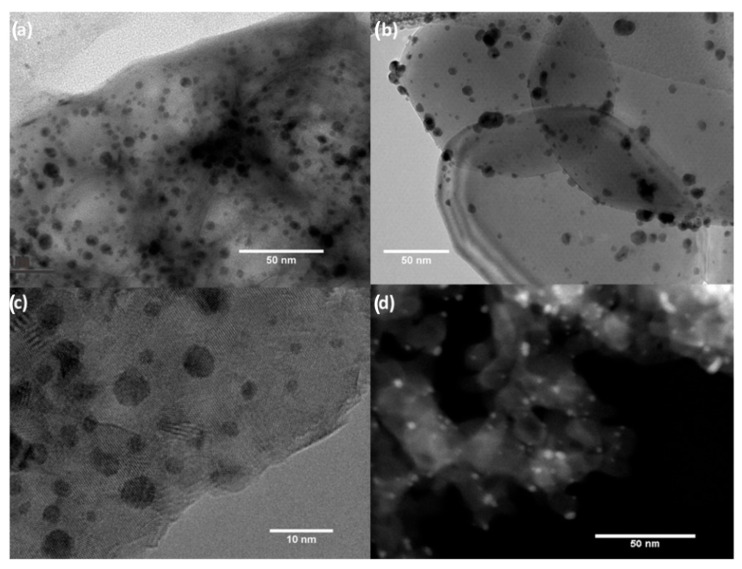
Transmission electron microscopy of (**a**) Au/MgO, (**b**) Au/SrO, (**c**) Au/CaO, and (**d**) Au/BaO dark field. Reprinted with permission from [42].

The Au-based catalysts in the cited studies were synthesized using the sol immobilization method. Initially, AuPVA (gold stabilized with polyvinyl alcohol, PVA) nanoparticles with an average size of about 3 nm were prepared, as confirmed by TEM [42]. Subsequently, these nanoparticles were dispersed onto various metal oxides. As expected, the size of the Au nanoparticles remained consistent when deposited on different metal oxides, with an average size of 4 ± 0.8 nm observed across all samples, as shown in Figure 4.

Considering the same Au content (2 wt.%) and comparable Au particle size, it was anticipated that the catalytic performance would primarily depend on the physicochemical properties and morphology of the support material [42].

**Scheme 3:**
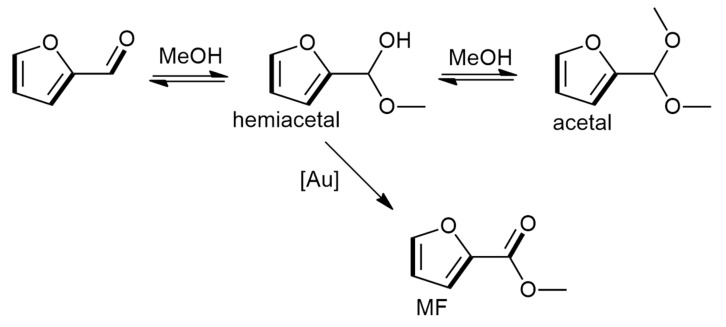
Reaction pathways for furfural oxidative esterification.

The synthesized gold catalysts were tested in the oxidative esterification of furfural in the liquid phase under air atmosphere. For this reaction, the presence of basic centers seems to be necessary, since only catalysts containing basic supports (MgO, CaO, BaO, NiO) were effective for oxidative esterification. The acetalization reaction is catalyzed by amphoteric and acidic supports such as TiO_2_, CeO_2_, and ZrO_2_. While the oxidation takes place on the metal surface, the support also plays a crucial role by forming more reactive intermediates. According to Casanova et al., the intermediate hemiacetal can be converted to either an acetal (acetalization) or an ester (oxidation), as illustrated in Scheme 3 [46]. Typically, hemiacetals are formed as intermediates, which are subsequently converted to acetals when considering the general mechanism of aldehyde reactivity. However, this applies specifically to esterification carried out under acidic conditions; under basic conditions, the hemiacetal remains in equilibrium with the initial aldehyde. The reactivity of various gold catalysts on basic oxides is the same. Upon the formation of hemiacetal, the catalyst with basic active centers predominantly produces MF (methylfuran) since acetal formation is hindered [46]. However, catalysts with acidic active centers facilitate both pathways, and the high yield of furfural acetal suggests a greater affinity for acetal formation over oxidation. Although a conversion from acetal to ester is possible, it is considered improbable. The presence of alkaline active centers facilitates furfural adsorption, leading to polarization of the H_aldehyde_–C bond. Subsequently, the electrophilic carbonyl group allows for nucleophilic attack by methanol (step 2). The basic center is then protonated, leading to the formation of an alkoxy group (step 3). A Au_alkoxy_ bond is established, facilitating β-hydrogenation (step 4). The regeneration of the catalyst in the presence of oxygen (stages 5–6) is extensively discussed by Zope et al. [47,48].

On the other hand, the presence of acid sites can also activate the H_aldehyde_–C bond, promoting nucleophilic attack by methanol. However, in this scenario, the formation of the Au_alkoxyl_ bond, which is crucial to this mechanism, seems unlikely. Once formed, the Au_alkoxy_ may have a higher tendency to bind to other acid centers rather than to Au [42].

**Figure 5:**
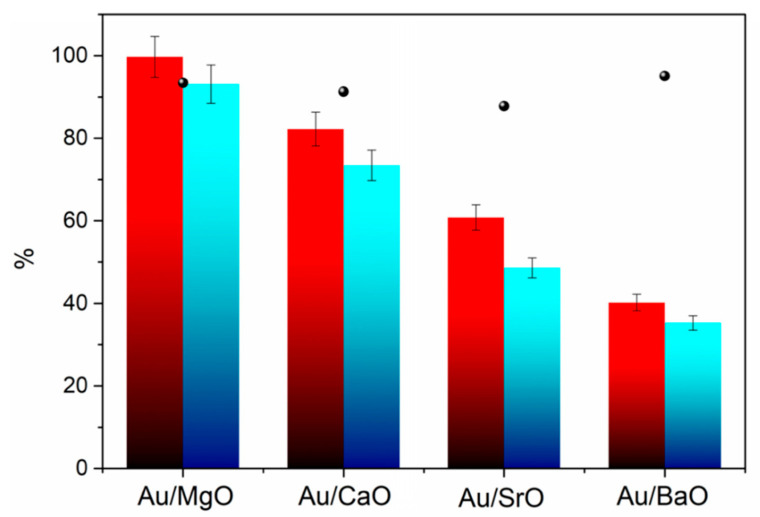
Oxidative esterification of furfural in methanol using Au catalyst. Conversion (■), methyl furoate (MF) yield (■), carbon balance (●). Conditions: furfural 24.7 mmol L^−1^, catalyst (catalyst:FFR:MeOH = 1:50:50,000), 2 h, 110 °C, 6-bar O_2_, 600 rpm. Reprinted with permission from [42].

Extensive investigations have been carried out involving gold catalysts supported on basic oxides. Notably, a trend of decreasing activity in the furfural oxidation reaction was observed as we moved downward in the alkaline earth group (Au/MgO > Au/CaO > Au/BaO) [41]. To further explore this trend, a catalyst based on strontium was also synthesized and tested. The results reaffirmed the earlier findings, with the Au/MgO catalyst exhibiting the highest activity in this transformation (Figure 5). Interestingly, under these conditions, the formation of acetals was not observed. These outcomes suggest that the presence of basic active centers in these oxides plays a crucial role in directing the reaction towards esters. Subsequent studies with Au/MgO revealed an even higher reaction rate, achieving complete conversion within just 30 min with exceptional MF yields (>90%). Additionally, concerning the substrate-to-metal ratio, higher FFR:Au ratios could be employed to achieve high MF yields, while a decrease in activity was observed for FFR:Au ratios exceeding 300. However, regardless of the ratios tested, the selectivity remained high, highlighting the specificity of this transformation [41].

The exceptional activity of the gold catalyst in furfural oxidation using methanol served as a foundation for investigating the oxidative esterification reaction with other alcohols featuring long and branched carbon chains. In the presence of these alcohols, the reaction proceeded at a slower rate, even when the FFR:Au ratio was lowered to 50 (Table 1). Nevertheless, successful oxidative esterification was achieved with ethanol (EtOH, Table 1, entry 1), isopropanol (*i*-PrOH, Table 1, entry 2), n-butanol (*n*-ButOH, Table 1, entry 3), and *iso*-pentanol (*i*-PentOH, Table 1, entry 4), leading to the formation of the corresponding esters (ethyl, *iso*-propyl, *n*-butyl, and *iso*-pentyl furoates) [42].

The resulting esters exhibited remarkable selectivity (>99%), with linear and long-chain alcohols yielding the highest ester yields. For instance, *n*-butyl furoate was produced with a remarkable 92% yield. It is noteworthy that branched-chain alcohols presented greater steric hindrance compared to linear alcohols, thereby making esterification in such cases more challenging. Additionally, long-chain alcohols allowed for higher conversion rates, although they displayed lower nucleophilicity when compared to short-chain alcohols [42].

## 4. Furfural Oxidation on Non-Basic Supports

Previous research suggests that the presence of a base or a basic support enhances the reaction rate, substrate concentration, and product solubility, and reduces product adsorption on the catalyst surface. However, in liquid-phase oxidation, the interface between gold particles and the support can become the site of reaction. Consequently, the activation energy may vary depending on the nature of these interactions. Metal–support interactions can influence the electronic properties of Au particles, potentially leading to slower reaction rates compared to unsupported Au nanoparticles. The extent of these interactions depends on the nature and structure of the support [49]. Therefore, it is essential to study the carrier’s effect without the use of base conditions. An investigation was conducted on furfural oxidation without the addition of a base, utilizing various manganese oxide (MnO_2_) structures as supports [50,51,52]. The goal was to establish a correlation between the support’s structure interacting with gold nanoparticles and their catalytic activity and selectivity. It is anticipated that achieving highly dispersed gold nanoparticles on MnO_2_ could enhance the mobility of lattice oxygen and weaken the bonds within the metal oxide. As a result, lattice oxygen or mobile oxygen may be released, thereby contributing to improved catalytic properties [53,54,55,56]. The authors of [50,51] employed a strategic approach to prepare ultra-dispersed gold nanoparticles of a similar medium size on various supports. The method involved using polyvinylpyrrolidone (PVP) as a stabilizer and NaBH_4_ as a reductant, a well-established technique for synthesizing metallic nanoparticles [57,58]. Three types of catalysts were synthesized: Au/MnO_2_-NF (nano flowers), Au/MnO_2_-NW (nano wires), and Au/MnO_2_-Comm (commercial MnO_2_ material) [50].

**Figure 6:**
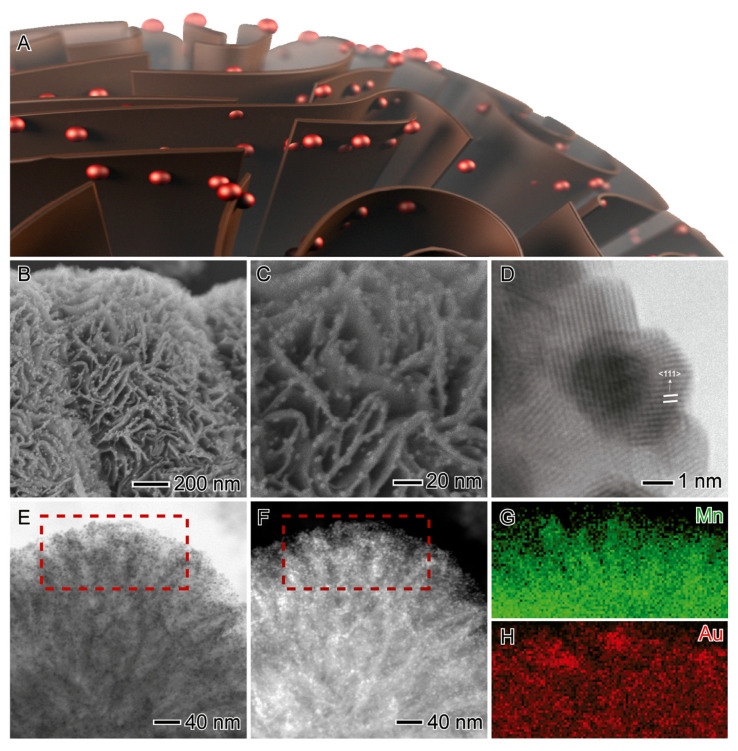
(**A**) Scheme depicting the MnO_2_ nanoflowers decorated with ultrasmall Au NPs at their surface and the corresponding SEM (**B**,**C**), HRTEM (**D**), STEM (**E**), and STEM-HAADF (**F**) images for the MnO_2_-Au nanoflowers. (**G**,**H**) STEM-EDX elemental maps for Mn (**G**) and Au (**H**) obtained from the regions indicated by dashed squares in (**E**,**F**). Reprinted with permission from [51].

The resulting gold nanoparticles (Au NPs) deposited on these different supports exhibited monodispersity and small size, with an average of approximately 2.6 nm (±0.8 nm) across all cases. Additionally, the Au nanoparticles were uniformly dispersed on the MnO_2_ surface, and no significant agglomeration of the gold nanoparticles was observed. Multi-technics microscopy images of the Au/MnO_2_-NF catalyst are presented in Figure 6, with SEM, HRTEM, STEM, and STEM-HAADF analyses.

Distinctly different morphologies were observed for the three different MnO_2_ carriers on TEM microscopic images (Figure 6). Furthermore, significant variations in the size of the specific surface area were also evident among these materials, as illustrated in Table 2.

**Figure 7:**
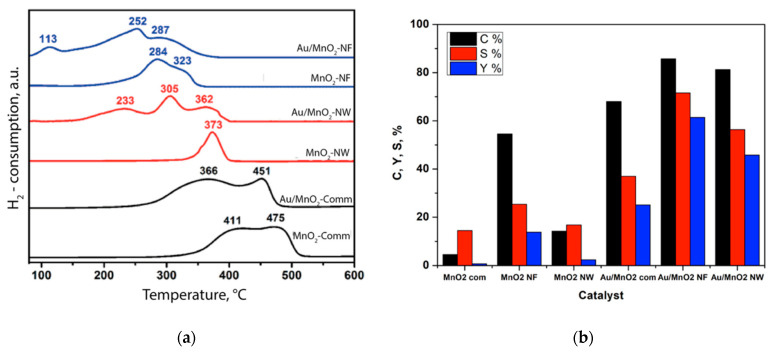
(**a**) Temperature-programmed reduction (TPR) profiles for MnO_2_ nanoflowers (in blue), nanowires (in red), and commercial MnO_2_ (in black) before and after the deposition of Au nanoparticles on their surfaces. (**b**) Furfural oxidation on different MnO_2_ oxides and Au-modified samples. *p* = 12 bar of air, temp. 110 °C, reaction time 2 h, FFR: Au molar ratio of 100. Reprinted with permission from [50].

Fascinating insights were obtained from H_2_-TPR studies (Figure 7a). The reducibility of the MnO_2_ support plays a crucial role in catalytic applications, as gold nanoparticles can strongly interact with metal oxides, altering their redox properties, which, in turn, impacts catalytic activity. The TPR profile for MnO_2_ nanoflowers exhibited two maxima at 284 and 323 °C, indicating the reduction of MnO_2_ to Mn_x_O_y_ with lower oxidation levels [59]. However, upon the deposition of ultrasmall Au NPs onto the surface of the nanoflowers, a distinct change in the TPR profile was observed, as illustrated in Figure 6A.

The reduction of MnO_2_ was observed at lower temperatures, revealing two main maxima corresponding to the reduction of Mn_2_O_3_ to Mn_3_O_4_ (252 °C), and MnO_2_ to Mn_2_O_3_ (287 °C) [60]. The low temperature peak observed at 113 °C is due to the gold reduction. Indeed, the XPS studies performed for these catalysts demonstrated the presence of oxidized gold at the surface. Specifically, the Au^δ+^ species ratio was ~26% in Au/MnO_2_-NF, which is a much higher proportion in comparison with the other supports. This could also be responsible for the enhanced catalytic activity of this catalyst. All prepared samples and pure supports were tested in the oxidation of furfural to furoic acid (Figure 7b). The pure supports themselves exhibited considerable activity in this reaction, achieving up to 55% conversion of furfural in the case of MnO_2_-NF. However, the selectivity to furoic acid was much lower in comparison to the gold-modified samples, reaching only about 25%. For example, the MnO_2_-NF sample achieved a stable furfural conversion of 55%, yet the selectivity to the acid was only 25%. In contrast, after just 2 h of reaction, the Au/MnO_2_-NF catalyst achieved an 86% furfural conversion (Figure 6B), while the gold catalyst deposited on commercial MnO_2_ showed only 69% conversion. Both catalysts (Au/MnO_2_-NF and Au/MnO_2_-NW) exhibited very high selectivity to furoic acid (over 80%), and the carbon balance values indicated no furfural degradation occurred. The activity of MnO_2_-based gold catalysts appears to strongly depend on the structure and morphology of the MnO_2_ used in the synthesis. However, it is not solely the differences in surface area and pore volume that account for the high activity of the MnO_2_-NF catalyst [50]. The deposition of Au NPs led to the formation of an increased number of oxygen vacancies or surface oxygen ions, attributed to the partial reduction of Mn^4+^ during the gold nanoparticle deposition step (as evident from XPS analysis). A significant amount of cationic Au^δ+^ forms upon the deposition of Au NPs was observed. Notably, in the Au/MnO_2_-NF sample, the proportion of Au^δ+^ forms reached 26% (only 18 and 20% were observed for Au/MnO_2_-Comm and Au/MnO_2_-NW, respectively). Previous studies on deposited Au nanoparticles have identified surface Au^δ+^ forms as the most catalytically active centers [61].

Developing catalysts for furfural oxidation poses a challenge in creating heterogeneous catalytic systems that maintain high activity and selectivity while eliminating the need for homogeneous bases. For gold catalysts, strategies to avoid alkali usage involve employing an alkaline support and/or introducing a second metal. In the latter approach, Au can form bimetallic nanomaterials with high selectivity, allowing the combination of both components’ advantages at the atomic level. This enhancement can significantly boost catalytic activity and stability during oxidation reactions of organic compounds in water [62].

Recently, new catalytic nanomaterials, named Au-Pd@SiTi, were developed by embedding AuPd nanoparticles within a titanium–silicate matrix. The study also investigated the effect of alloy enrichment with either gold or palladium on the catalytic activity and selectivity of these materials. The catalysts were prepared using previously synthesized nanoparticles, achieved through a well-known and reproducible method that produces Au, Pd, and Au-Pd nanoparticles of approximately 4 nm in size. These nanoparticles were stabilized with sodium citrate and then reduced using NaBH_4_ [62].

**Figure 8:**
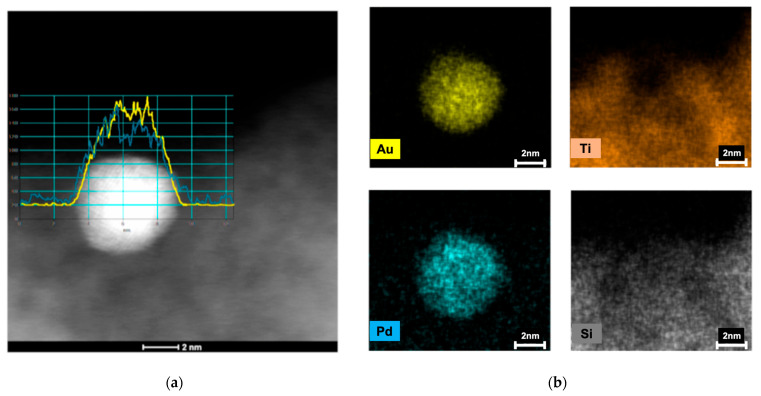
(**a**) HRTEM HAADF images of the Au1Pd1@SiTi catalyst and (**b**) X-ray mapping of the sample for Au, Ti, Pd, and Si. Reprinted with permission from [62].

The synthesized nanoparticles were immersed in a mixture of organo-metallic titanium and silicate precursors, and their size remained unchanged after immobilization in the carrier. The embedded Au1Pd1 nanoparticles displayed monodispersity, with an average size of 3.8 ± 0.8 nm (Figure 8a). Given the same metal loading (approximately 2.2 wt.%) and particle size, the catalytic properties were expected to be mainly influenced by the metal–support interaction and the chemical composition of the bimetallic nanoparticles [62]. The presence of Pd-rich or Au-rich nanorods could have a particularly significant impact on catalytic activity. High-resolution transmission electron microscopy (HRTEM, Thermo Fischer Scientific, Hillsboro, OR, USA) images and corresponding energy dispersive X-ray spectroscopy (EDS, Thermo Fischer Scientific, Hillsboro, OR, USA) analysis results for the Au1Pd1@SiTi catalyst are displayed in Figure 8b, confirming that the average size of gold nanoparticles remained unchanged after encapsulation in SiTi. Additionally, X-ray mapping revealed some segregation of Au and Pd in the nanoparticles. Catalytic tests for furfural oxidation were conducted using Au, Pd, and AuPd-titanosilicate catalysts, with high-pressure air (26 bar) as the oxidant. The results demonstrated that even in the absence of alkali, the catalysts effectively oxidized furfural to furoic acid (FA) (Table 3). However, in all cases, the carbon balance was relatively low, which can be attributed to furfural decomposition on acid supports and/or adsorption of furfural on the SiTi matrix. Nevertheless, the data presented in Table 3 underscored the synergistic performance of the bimetallic AuxPdy catalysts in comparison to their monometallic counterparts. Among them, Au4Pd1 and Au1Pd1 displayed the highest activity, with the former leading to greater acid synthesis yields. Notably, the Au4Pd1@SiTi catalyst exhibited a slightly higher yield (expressed in TON—turnover number) than the other systems [62].

The embedded systems exhibit a higher level of intermolecular interactions between the metallic nanoparticles and the oxide of the titanosilicate matrix compared to classical metal nanoparticles deposited on the oxide. The presented synthesis method of embedded catalysts enables the maintenance of high metal dispersion, even at relatively high metal content. The degree of interactions between the carrier and the metal can be controlled, leading to various stabilization mechanisms. The close interaction between gold and the titania in embedded materials significantly enhances the catalytic activity in base-free furfural oxidation. However, in this case, controlling the gold particle size and dispersion is quite challenging. To address this issue, a new type of catalyst based on an inverse core–shell structure has been studied [63,64].

**Figure 9:**
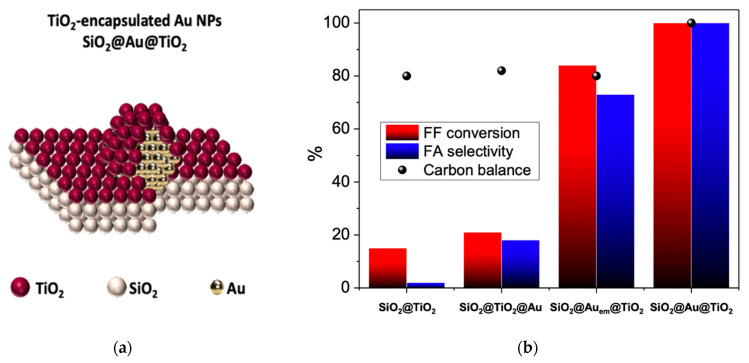
Furfural oxidation on supported and encapsulated Au catalysts: (**a**) schematic representation of the TiO_2_-encapsulated Au nanoparticles; (**b**) catalytic results. Reprinted with permission from [64].

The formation of the TiO_2_ shell on the SiO_2_ surface was achieved through the intermediate formation of titanium hydroxide (Ti(OH)_4_), and Au nanoparticles were partially embedded into TiO_2_ (Figure 9a). This synthesis method resulted in the creation of core–shell particles, where a homogeneous TiO_2_ shell with an average thickness of 3 nm encapsulated the AuNPs. The mean size of the Au nanoparticles, calculated from TEM micrographs, was approximately 5 nm for each catalyst. Figure 9b displays the conversions achieved in the base-free oxidation of FFR in water using air as the oxidant with the three Au catalysts under similar conditions. A significant enhancement in catalytic activity was observed for the SiO_2_@Au_em_@TiO_2_ catalyst, where the TiO_2_ support partially embeds the AuNPs, indicating the crucial role of the Au–TiO_2_ interface. The catalyst with TiO_2_-encapsulated AuNPs exhibited remarkable catalytic activity (100% selectivity at 100% conversion) compared to its supported and embedded counterparts (Figure 9), with no by-products originating from side reactions detected during the catalytic cycle [64]. Encouragingly, a 100% carbon balance was achieved with this catalyst. Furthermore, the influence of Au loading on the catalytic behavior of the SiO_2_@Au@TiO_2_ core–shell catalyst was investigated. In contrast to the supported catalysts, the core–shell materials demonstrated that total and selective conversion of FFR to FA could be achieved even at low Au loadings, as low as 0.25 wt.% [64].

## 5. Parameters Influencing Base-Free Furfural Oxidation: Machine Learning Study

To the best of our knowledge, this paper represents the first comprehensive study that has gathered experimental data to evaluate the influence of catalyst predictors on catalyst performance. A total of 93 catalysts, discussed earlier, were collected from recent research conducted in the same laboratory between 2018 and 2022, all under the same reaction conditions. These catalysts were synthesized for the oxidation of furfural to furoic acid in aqueous media. A machine learning (ML) approach was employed to assess the most influential factor during the catalyst optimization process. This ML approach analyzes, interprets and structures the data between the reaction conditions and the catalyst characteristics as predictor variables, and the yield of the oxidation reaction of FFR to FA as the response variable. Due to the high cost of the catalyst and its development, another response should be considered, that is, the catalyst stability or lifetime represented by the cycle number.

The Minitab software (Minitab®, v21.2 64-bit) was used to perform the machine learning analysis and produce associated graphics. The results are presented as an optimal tree diagram that illustrates important patterns and relationships between a continuous response and important predictors by means of nods and leaves within 1 standard error of the maximum R-squared. Also, the relative variable importance is used, providing relative ranking information about how many variables to control or monitor for the catalyst or the reaction optimization.

Table 4 provides a comprehensive summary of the catalysts used in this study, while Table 5 presents crucial reaction condition data. These datasets serve as predictors, as they significantly influence the catalytic performances of the catalysts under investigation. The response variable utilized for comparing the catalysts’ effectiveness was the yield of FA. Also, the number of recycling tests or leaching was used as a response to study the lifetime of the catalyst.

In this study, various parameters were considered as important predictors for the catalytic oxidation reactions. The type of catalyst, that is, whether it is monometallic or bimetallic, holds relevance for this reaction. It was observed that a metal loading larger than 2 wt.% can lead to the formation of a bulk active phase instead of dispersed nanoparticles, significantly impacting the conversion. Hence, metal loading and metal particle size were also considered as relevant predictors. The nature of the catalyst support used in the catalyst synthesis is one of the most crucial parameters. The support not only aids in dispersing the active phase but also provides basic active sites. Given the high cost involved in catalyst development, the catalyst’s stability or lifetime, represented by the number of reuse cycles, should also be taken into account as an important response or a predictor for the FA yield study.

As discussed earlier, noble metals have shown remarkable effectiveness as the active phase for base-free oxidation reactions. Monometallic gold-supported catalysts, in particular, have demonstrated high selectivity and stability in furfural oxidation. However, the addition of a second noble metal, such as Pd or Pt, to form a bimetallic catalyst can significantly impact the selectivity and the overall oxidation reaction mechanism. Furthermore, the type of support, its synthesis method, and its chemical characteristics are all considered relevant parameters and are included in Table 4 for further analysis. The reaction conditions significantly influence the selectivity in furfural oxidation. Table 5 encompasses various factors considered in this study, such as the substrate/metal (mol/mol) ratio (ranging from 50 to 100), reaction temperature (ranging from 80 to 110 °C), reaction pressure (ranging from 1 to 26 bar), reaction time (ranging from 2 to 10 h), and pH of the reaction medium (ranging from 3 to 10.5). A total of 15 predictors were examined using a machine learning approach to estimate their effects on furoic acid yield and the number of reaction cycles as responses.

**Figure 10:**
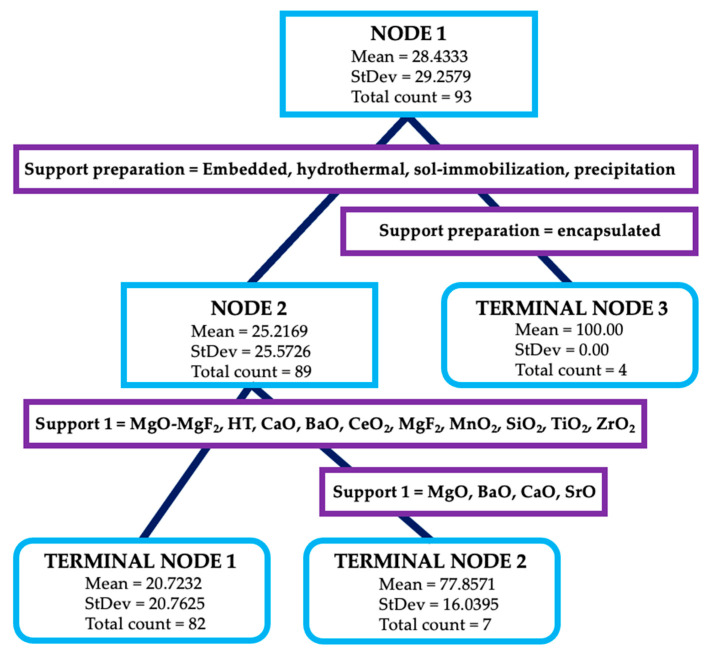
Optimal tree diagram with 15 predictors and the yield as response.

The machine learning algorithm used in this study is the decision tree known as a regression tree (CART). This approach identifies important variables and groups in the data with desirable characteristics and can be applied to both continuous and categorical data. The regression tree builds a regression model in the form of a tree structure, composed of root nodes (the training dataset), inner nodes (acting as decision nodes, splitting based on the best feature), and leaf nodes (making decisions). When considering all 93 catalysts with yield as the response, the decision tree shows a mean yield of 28.43% in root node 1 (Figure 10). The root node is divided into two nodes using k-fold cross-validation, and the catalyst preparation method acts as the splitter, resulting in terminal node 3 and decision node 2. Terminal node 3 reveals that for catalysts prepared using the encapsulated method, the yield is 100 for four catalysts, demonstrating the significance of catalyst preparation for the oxidation reaction. The encapsulation method guarantees a good stability and activity of the catalyst. The CART algorithm seeks the best homogeneity in response for the sub-nodes. Node 2 is then split based on the type of support used. This predictor seems to be the most relevant one, as changing the support implies a shift in the pH of the reaction medium.

When using certain supports such as hydrotalcite, MnO_2_, BeO, CeO_2_, MgF_2_, SiO_2_, TiO_2_, and ZrO_2_, the pH of the oxidation reaction medium is lower than 7.7. The corresponding mean yield in terminal node 1 for these supports is approximately 20.72%. On the other hand, bifunctional supports like MgO-MgF_2_, BaO, CaO, and SrO lead to a basic pH > 7.8, resulting in a higher mean yield in terminal node 2 (about 77.8%). These results highlight the relationship between the support catalyst and the pH of the reaction medium. When the pH is neutral or acidic, a low yield is observed, while a high pH leads to a higher yield of furoic acid. These findings underscore the importance of optimizing the support as a key parameter for enhancing the yield. No additional parameter emerges in the decision tree analysis. This reaffirms, once again, the importance of the support’s influence on the yield of this oxidation reaction.

**Figure 11:**
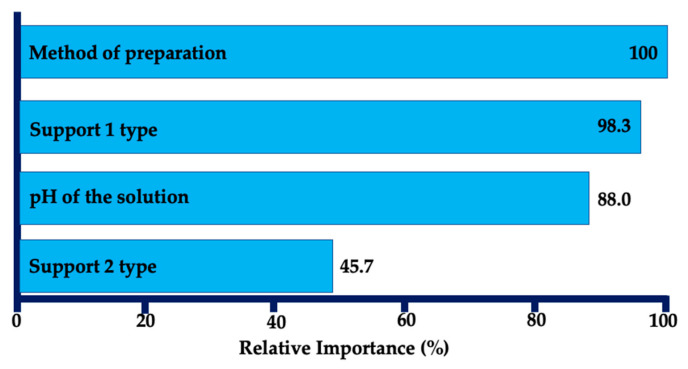
Relative importance variable for the furoic acid yield.

Figure 11 presents the results of the tree, ranked according to the relative importance of the variables for the yield. This diagram highlights which variables should be the focus to enhance the yield. The variables are listed in order of importance, with the most crucial predictor at the top and the least significant at the bottom. The importance of variables quantifies the model’s improvement when splits are made on a particular predictor. Relative importance is expressed as the percentage improvement over the most significant predictor. Consistent with the decision tree findings, the main effect identified is the preparation of the catalyst support, then the support type, followed by the solution pH, which is determined by the chemical nature of the supports (pH being dependent on leaching extent). This result strongly emphasizes that the catalyst support plays the most vital role in determining the success of this reaction when using noble metals in an aqueous medium. Understanding and optimizing the support can significantly impact the yield and efficiency of furfural oxidation.

**Figure 12:**
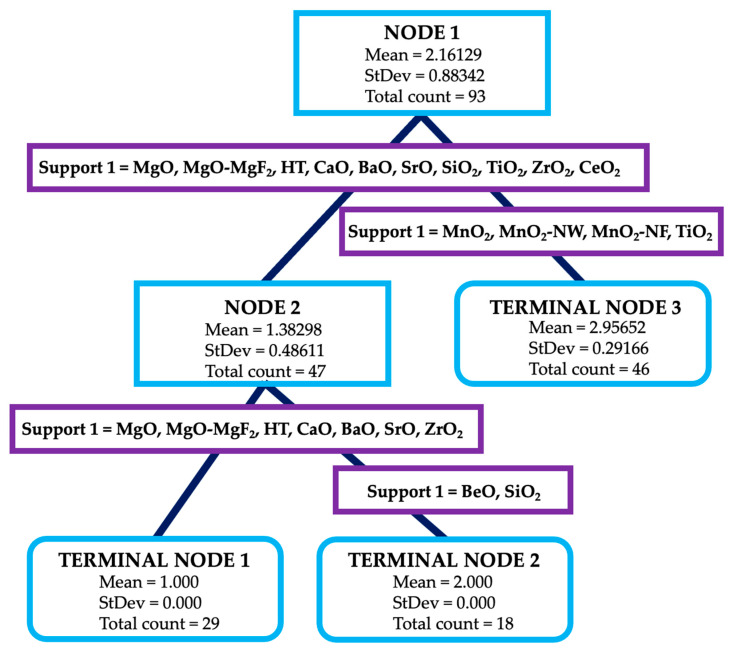
Optimal tree diagram with 15 predictors and the number of cycles as response.

In the analysis of the predictors’ effect on the number of cycles or catalyst leaching, the decision tree yields similar insights. The most significant predictor, once again, is the support used for the catalyst. For this investigation, we sought the predictor that would increase the number of cycles, indicating a more stable catalyst. In Figure 12, root node 1 is split into two sub-nodes based on the nature of the support. When the support is soluble in water, leaching occurs, restricting the number of cycles to one, rendering the catalyst non-recyclable. This limitation applies to catalysts supported on MgO, CaO, BaO, hydrotalcite, and MgO-MgF_2_ due to their inherent basicity and leaching tendency in an aqueous medium. Terminal node 1 has a mean value of one, signifying that these catalysts lose stability after the first test and are not practically viable for commercial use. On the other hand, node 2 indicates that SiO_2_ and BeO show more promising results with reduced leaching. Terminal node 3 of this decision tree provides clear evidence that using TiO_2_ and MnO_2_ as supports is the most promising, as it allows for at least three recycling cycles, making it a favorable option for stable and recyclable catalysts.

**Figure 13:**
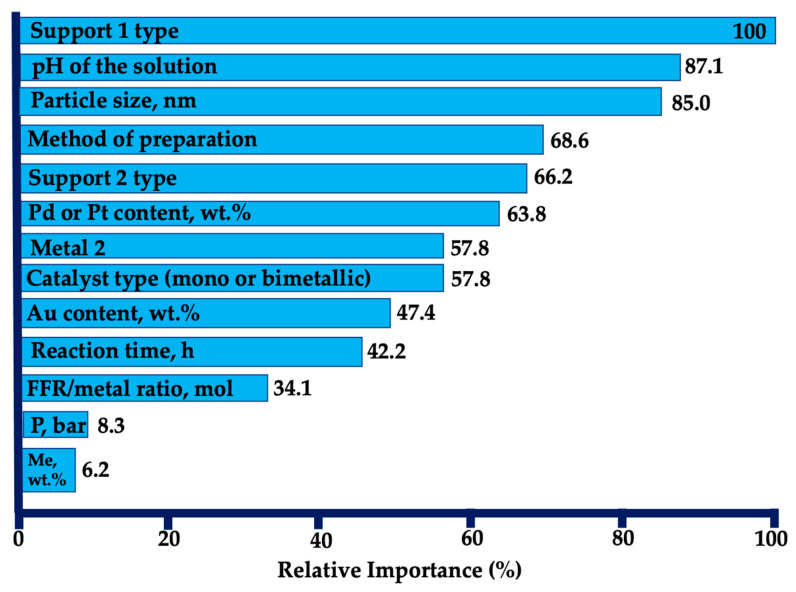
Relative importance variable for the recycle studies (stability of the catalysts).

Regarding the relative importance of the variables, the diagram ranks the predictors in increasing order of their impact on increasing the number of cycles (Figure 13). Support 1, which refers to the catalyst support, emerges as the primary predictor to focus on for optimizing and preventing leaching. The pH solution that is connected to the support type seems to affect the leaching up to 97.9%.

Additionally, the contents of Pt and Pd (noble metals) and particle size exhibit over 80% positive effects in increasing the number of cycles, making them significant factors to consider for enhancing catalyst stability and recyclability.

## 6. Conclusions

The findings of this study showcase the feasibility of synthesizing furoic acid in non-controlled acidic solutions, as presented through various publications. The support material used in gold-based catalysts has been identified as a critical factor, and exceptional results were achieved with basic supports like MgO, CaO, BaO, and hydrotalcites. The in situ formation of OH^−^ ions from hydrotalcites and MgO during the reaction was observed in the case of basic supports. Additionally, a novel binary catalyst, MgF_2_–MgO, demonstrated minimized MgO leaching and enabled high yields of furoic acid even at a pH of 3.6.

Moreover, the high activity of SiO_2_@TiO_2_ supports in furfural oxidation with gold catalysts was demonstrated. These supports facilitated the reaction at low pH with enhanced selectivity, overcoming the limitations of product adsorption. The approach was further extended to investigate core–shell-structured heterogeneous catalysts, leveraging strong support–metal interactions (SMSIs). Preliminary studies indicated remarkable activity and selectivity of SiO_2_@Au@TiO_2_ materials.

The application of machine learning proved to be a powerful tool in analyzing close to 100 heterogeneous catalysts. This study showcased its usefulness in comparing published data for a specific reaction. In certain cases, comparable data were not readily available, such as information on the carbon balance, solution pH, or ICP analysis, which emphasized the importance of comprehensive data reporting in published articles.

The field of base-free oxidation of furfural, as with many areas of research, is likely to continue evolving and experiencing new trends in the future. The potential future trends in this field mostly concern catalyst development, mechanistic understanding, and flow reactor development. Focus should be put on designing and synthesizing new catalysts with enhanced selectivity, activity, and stability. This may involve exploring advanced nanomaterials, metal–organic frameworks (MOFs), and other innovative catalyst structures. As discussed in this review article, the most promising materials are based on encapsulated gold nanoparticles. A deeper understanding of the reaction mechanisms and intermediates involved in furfural oxidation is expected. Advances in analytical techniques and computational methods will contribute to uncovering reaction pathways. In particular, we foresee the development of new operando or in situ techniques that permit the study of liquid-phase reactions, not only focusing on the catalyst structure and composition but also on the formation of intermediates or adsorbed species. An example could be the use of in situ EPR studies of the radicals formed during the oxidation with gold. A trend observed for another type of reaction is the development of continuous-flow chemistry technology. This has the potential to become a new trend in the field of base-free oxidation of furfural as it offers several advantages that could benefit research in this field. Complex furfural oxidation processes involving multiple reaction steps can be performed sequentially in a single flow system. Finally, extremely interesting, from an industrial point of view, could be the continuous production of furoic acid (or maleic, if necessary) directly from lignocellulosic wastes through extraction of hemicellulose, depolymerization, and oxidation.

## Figures and Tables

**Table 1:** Oxidative esterification of furfural in various alcohols using Au/MgO catalyst. Experimental conditions: FFR 24.7 mmol L^−1^, Au/MgO (Au:FFR:alcohol = 1:50:50,000), 15 h, 110 °C, 6-bar O_2_, 600 rpm. Adapted with permission from [42].

Entry	Substrate/Solvent	FFR Conversion (%)	Yield (%)
1	ethanol	45	45
2	*iso*-propanol	32	31
3	*n*-butanol	92	92
4	*iso*-pentanol	68	66

**Table 2:** Au wt.%, Au gold particle size, BET surface area, pore volume and pore diameter determined with FAAS, TEM, and BET-N_2_, respectively. Reprinted with permission from [50].

Catalysts	Au [%]	Particle Size [nm]	Surface Area [m^2^/g]	Pore Volume [cm^3^/g]	Pore Diameter [A]
Au/MnO_2_-NF	1.3	2.6	196	0.42	6.1
MnO_2_-NF	-	-	194	0.41	6.1
Au/MnO_2_-NW	1.3	3.2	125	0.16	6.9
MnO_2_-NW	-	-	122	0.16	6.8
Au/MnO_2_-Comm	1.4	2.6	15	0.05	17.8
MnO_2_-Comm	-	-	14	0.05	17.8

**Table 3:** Base-free oxidation of furfural for 10 h reaction using embedded AuxPdy@SiTi and supported Au and Pd catalysts. (Conditions: FFR/metal ratio = 50 (mol/mol), air = 26 bar, 110 °C, 600 rpm.) Reprinted with permission from [62].

Catalysts	Conversion [%]	Selectivity [%]	Yield [%]	TON	Carbon Balance [%]
Au_4_Pd_1_@SiTi	83	45	37	91	54
Au_1_Pd_1_@SiTi	77	30	23	80	47
Au_1_Pd_4_@SiTi	54	23	12	57	58
Au_4_Pd_1_/SiTi	99	48	48	50	49
Au_1_Pd_1_/SiTi	54	18	10	28	56
Au_1_Pd_4_/SiTi	38	11	4	19	66
Au_1_Pd_1PVA_@SiTi	87	41	36	44	48
Au/TiO_2_	100	8	8	43	8
Au/ZrO_2_	92	32	29	45	37
blank	52	0	0	-	48
Au@SiTi	30	32	10	15	80
Pd@SiTi	34	20	7	17	73

**Table 4:** Catalysts used for the machine learning studies. In total, 93 catalysts were chosen.

Catalyst	Metal 1	Metal 2	Supp. 1	Supp. 2	Synthesis Method	Au wt.%	Pd or Pt wt.%	Metal wt.%	Particle Size [nm]	FA Yield [%]	Ref.
Au4Pd1@SiTi	Au	Pd	SiO_2_	TiO_2_	Embedded	1.68	0.42	2.11	4	37	[62]
Au1Pd1@SiTi	Au	Pd	SiO_2_	TiO_2_	Embedded	1..37	0.97	2.34	3.8	23	[62]
Au1Pd4@SiTi	Au	Pd	SiO_2_	TiO_2_	Embedded	0.69	1.6	2.29	4	12	[62]
Au4Pd1/SiTi	Au	Pd	SiO_2_	TiO_2_	Embedded	5.41	0.8	6.21	4	48	[62]
Au1Pd1/SiTi	Au	Pd	SiO_2_	TiO_2_	Embedded	2.3	1.31	3.61	3.8	10	[62]
Au1Pd4/SiTi	Au	Pd	SiO_2_	TiO_2_	Embedded	1.21	2.21	3.34	4	4	[62]
Au@SiTi	Au	No	SiO_2_	TiO_2_	Embedded	6.4	0	6.4	4	10	[62]
Pd@SiTi	Pd	No	SiO_2_	TiO_2_	Embedded	0	3.1	3.1	4	7	[62]
HT-4:1	No	No	0.8 MgO	0.2 Al_2_O_3_	Precipitation	0	0	0	0	1	[40]
Au/HT-4:1	Au	No	0.8 MgO	0.2 Al_2_O_3_	Precipitation	1.64	0	1.64	3.5	9	[40]
Au/HT-H_2_O_2_-4:1	Au	No	0.8 MgO	0.2 Al_2_O_3_	Precipitation	1.64	0	1.64	3.5	19	[40]
Au/HT-4:1	Au	No	0.8 MgO	0.2 Al_2_O_3_	Precipitation	1.64	0	1.64	3.5	27	[40]
HT-2:1	No	No	0.6 MgO	0.4 Al_2_O_3_	Precipitation	0	0	0	0	0	[40]
Au/HT-2:1	Au	No	0.6 MgO	0.4 Al_2_O_3_	Precipitation	1.52	0	1.52	3.5	6	[40]
Au/HT-H_2_O_2_-2:1	Au	No	0.6 MgO	0.4 Al_2_O_3_	Precipitation	1.52	0	1.52	3.5	17	[40]
Au/HT-2:1	Au	No	0.6 MgO	0.4 Al_2_O_3_	Precipitation	1.52	0	1.52	3.5	20	[40]
HT-1:1	No	No	0.5 MgO	0.5 Al_2_O_3_	Precipitation	0	0	0	0	0	[40]
Au/HT-1:1	Au	No	0.5 MgO	0.5 Al_2_O_3_	Precipitation	1.83	0	1.83	3.5	6	[40]
Au/HT-H_2_O_2_-1:1	Au	No	0.5 MgO	0.5 Al_2_O_3_	Precipitation	1.83	0	1.83	3.5	22	[40]
Au/HT-1:1	Au	No	0.5 MgO	0.5 Al_2_O_3_	Precipitation	1.83	0	1.83	3.5	25	[40]
HT-1:5	No	No	0.2 MgO	0.8 Al_2_O_3_	Precipitation	0	0	0	0	0	[40]
Au/HT-1:5	Au	No	0.2 MgO	0.8 Al_2_O_3_	Precipitation	1.9	0	1.9	3.5	0	[40]
Au/HT-H_2_O_2_-1:5	Au	No	0.2 MgO	0.8 Al_2_O_3_	Precipitation	1.9	0	1.9	3.5	5	[40]
Au/HT-1:5	Au	No	0.2 MgO	0.8 Al_2_O_3_	Precipitation	1.9	0	1.9	3.5	3	[40]
Au/TiO_2_	Au	No	TiO_2_	No	Sol-PVA ^a^	2.09	0	2.09	3	40.48	[65]
Pt/TiO_2_	Pt	No	TiO_2_	No	Sol-PVA ^a^	0	0.91	0.91	3	0.99	[65]
Pd/TiO_2_	Pd	No	TiO_2_	No	Sol-PVA ^a^	0	1.98	1.98	3	1.45	[65]
0.5%Pt1-Au1/TiO_2_	Au	Pt	TiO_2_	No	Sol-PVA ^a^	0.24	0.16	0.4	3	21.8	[65]
1.25%Pt1-Au1/TiO_2_	Au	Pt	TiO_2_	No	Sol-PVA ^a^	0.62	0.42	1.04	3	30.1	[65]
2%Pt1-Au1/TiO_2_	Au	Pt	TiO_2_	No	Sol-PVA ^a^	1.02	0.75	1.77	3	24.9	[65]
0.5%Pt1-Au3/TiO_2_	Au	Pt	TiO_2_	No	Sol-PVA ^a^	0.37	0.12	0.49	3	23.6	[65]
1.25%Pt1-Au3/TiO_2_	Au	Pt	TiO_2_	No	Sol-PVA ^a^	0.9	0.29	1.18	3	32.7	[65]
2%Pt1-Au3/TiO_2_	Au	Pt	TiO_2_	No	Sol-PVA ^a^	1.36	0.43	1.79	3	37.5	[65]
0.5%Pt3-Au1/TiO_2_	Au	Pt	TiO_2_	No	Sol-PVA ^a^	0.13	0.19	0.32	3	15.2	[65]
1.25%Pt3-Au1/TiO_2_	Au	Pt	TiO_2_	No	Sol-PVA ^a^	0.26	0.5	0.77	3	22.8	[65]
2%Pt3-Au1/TiO_2_	Au	Pt	TiO_2_	No	Sol-PVA ^a^	0.5	1.19	1.69	3	15.6	[65]
0.5%Pd1-Au1/TiO_2_	Au	Pd	TiO_2_	No	Sol-PVA ^a^	0.31	0.18	0.49	3	22.8	[65]
1.25%Pd1-Au1/TiO_2_	Au	Pd	TiO_2_	No	Sol-PVA ^a^	0.89	0.48	1.37	3	29.9	[65]
2%Pd1-Au1/TiO_2_	Au	Pd	TiO_2_	No	Sol-PVA ^a^	1.29	0.68	1.97	3	34.5	[65]
0.5%Pd1-Au3/TiO_2_	Au	Pd	TiO_2_	No	Sol-PVA ^a^	0.4	0.09	0.49	3	19.8	[65]
1.25%Pd1-Au3/TiO_2_	Au	Pd	TiO_2_	No	Sol-PVA ^a^	1.15	0.22	1.37	3	31.5	[65]
2%Pd1-Au3/TiO_2_	Au	Pd	TiO_2_	No	Sol-PVA ^a^	1.59	0.29	1.88	3	34.1	[65]
0.5%Pd3-Au1/TiO_2_	Au	Pd	TiO_2_	No	Sol-PVA ^a^	0.17	0.3	0.47	3	11.4	[65]
1.25%Pd3-Au1/TiO_2_	Au	Pd	TiO_2_	No	Sol-PVA ^a^	0.45	0.72	1.17	3	14.8	[65]
2%Pd3-Au1/TiO_2_	Au	Pd	TiO_2_	No	Sol-PVA ^a^	0.78	1.21	1.99	3	17.6	[65]
0.5%Pt1-Au1/TiO_2_	Au	Pt	TiO_2_	No	Sol-PVA ^a^	0.24	0.16	0.4	3	8.8	[65]
1.25%Pt1-Au1/TiO_2_	Au	Pt	TiO_2_	No	Sol-PVA ^a^	0.62	0.42	1.04	3	0	[65]
2%Pt1-Au1/TiO_2_	Au	Pt	TiO_2_	No	Sol-PVA ^a^	1.02	0.75	1.77	3	0	[65]
0.5%Pt1-Au3/TiO_2_	Au	Pt	TiO_2_	No	Sol-PVA ^a^	0.37	0.12	0.49	3	28.8	[65]
1.25%Pt1-Au3/TiO_2_	Au	Pt	TiO_2_	No	Sol-PVA ^a^	0.9	0.29	1.18	3	0.9	[65]
2%Pt1-Au3/TiO_2_	Au	Pt	TiO_2_	No	Sol-PVA ^a^	1.36	0.43	1.79	3	0	[65]
0.5%Pt3-Au1/TiO_2_	Au	Pt	TiO_2_	No	Sol-PVA ^a^	0.13	0.19	0.32	3	5.2	[65]
1.25%Pt3-Au1/TiO_2_	Au	Pt	TiO_2_	No	Sol-PVA ^a^	0.26	0.5	0.77	3	0	[65]
2%Pt3-Au1/TiO_2_	Au	Pt	TiO_2_	No	Sol-PVA ^a^	0.5	1.19	1.69	3	0	[65]
0.5%Pd1-Au1/TiO_2_	Au	Pd	TiO_2_	No	Sol-PVA ^a^	0.31	0.18	0.49	3	33.5	[65]
1.25%Pd1-Au1/TiO_2_	Au	Pd	TiO_2_	No	Sol-PVA ^a^	0.89	0.48	1.37	3	24.5	[65]
2%Pd1-Au1/TiO_2_	Au	Pd	TiO_2_	No	Sol-PVA ^a^	1.29	0.68	1.97	3	13.2	[65]
0.5%Pd1-Au3/TiO_2_	Au	Pd	TiO_2_	No	Sol-PVA ^a^	0.4	0.09	0.49	3	51.6	[65]
1.25%Pd1-Au3/TiO_2_	Au	Pd	TiO_2_	No	Sol-PVA ^a^	1.15	0.22	1.37	3	58.2	[65]
2%Pd1-Au3/TiO_2_	Au	Pd	TiO_2_	No	Sol-PVA ^a^	1.59	0.29	1.88	3	58.7	[65]
0.5%Pd3-Au1/TiO_2_	Au	Pd	TiO_2_	No	Sol-PVA ^a^	0.17	0.3	0.47	3	12.2	[65]
1.25%Pd3-Au1/TiO_2_	Au	Pd	TiO_2_	No	Sol-PVA ^a^	0.45	0.72	1.17	3	0.9	[65]
2%Pd3-Au1/TiO_2_	Au	Pd	TiO_2_	No	Sol-PVA ^a^	0.78	1.21	1.99	3	0	[65]
Au/MgO	Au	No	MgO	No	Sol-PVA ^a^	1.8	0	1.8	3.6	100	[41]
Au/TiO_2_	Au	No	TiO_2_	No	Sol-PVA ^a^	1.8	0	1.8	3.6	30	[41]
Au/ZrO_2_	Au	No	ZrO_2_	No	Sol-PVA ^a^	1.8	0	1.8	3.6	32	[41]
Au/CeO_2_	Au	No	CeO_2_	No	Sol-PVA ^a^	1.8	0	1.8	3.6	18	[41]
Au/MgF_2_	Au	No	MgF_2_	No	Precipitation	1.8	0	1.8	3.6	2	[41]
Au/0.6MgF_2_-0.4MgO	Au	No	0.4 MgO	0.6 MgF_2_	Precipitation	1.8	0	1.8	3.6	76	[41]
Au/0.4MgF_2_-0.6MgO	Au	No	0.6 MgO	0.4 MgF_2_	Precipitation	1.8	0	1.8	3.6	99	[41]
SiO_2_@TiO_2_	No	No	SiO_2_	TiO_2_	Sol-PVA ^a^	0	0	0	5	0.3	[64]
0.25% SiO_2_@Au@TiO_2_	Au	No	SiO_2_	TiO_2_	Encapsulated	0.13	0	0.13	5	100	[64]
0.5% SiO_2_@Au@TiO_2_	Au	No	SiO_2_	TiO_2_	Encapsulated	0.53	0	0.53	5	100	[64]
1% SiO_2_@Au@TiO_2_	Au	No	SiO_2_	TiO_2_	Encapsulated	1.13	0	1.13	5	100	[64]
2% SiO_2_@Au@TiO_2_	Au	No	SiO_2_	TiO_2_	Encapsulated	2.02	0	2.02	5	100	[64]
0.25% SiO_2_@TiO_2_@Au	Au	No	SiO_2_	TiO_2_	Embedded	0.28	0	0.28	5	8.4	[64]
0.5% SiO_2_@TiO_2_@Au	Au	No	SiO_2_	TiO_2_	Embedded	0.56	0	0.56	5	41.8	[64]
1% SiO_2_@TiO_2_@Au	Au	No	SiO_2_	TiO_2_	Embedded	1.26	0	1.26	5	19	[64]
2% SiO_2_@TiO_2_@Au	Au	No	SiO_2_	TiO_2_	Embedded	1.37	0	1.37	5	3.8	[64]
2%Au/CaO	Au	No	CaO	No	Sol-PVA ^a^	2	0	2	3.5	81	[66]
2%Au/SrO	Au	No	SrO	No	Sol-PVA ^a^	2	0	2	3.6	66	[66]
2%Au/BaO	Au	No	BaO	No	Sol-PVA ^a^	2	0	2	3.7	54	[66]
2%Au/BeO	Au	No	BeO	No	Sol-PVA ^a^	2	0	2	3.8	32	[66]
Au-HT2:1	Au	No	0.6MgO	0.4Al_2_O_3_	Precipitation	1.3	0	1.3	3.7	72	[38]
Au-HT3:1	Au	No	0.7MgO	0.3Al_2_O_3_	Precipitation	1.7	0	1.7	3.7	68	[38]
Au-HT4:1	Au	No	0.75MgO	0.25Al_2_O_3_	Precipitation	1.2	0	1.2	3.7	100	[38]
Au-HT5:1	Au	No	0.8MgO	0.2Al_2_O_3_	Precipitation	1.4	0	1.4	3.7	100	[38]
MnO_2_ com	No	No	MnO_2_	No	Hydrothermal	0	0	0	0	2	[50]
MnO_2_ NF	No	No	MnO_2_ NF	No	Hydrothermal	0	0	0	0	14	[50]
MnO_2_ NW	No	No	MnO_2_ NW	No	Hydrothermal	0	0	0	0	4	[50]
Au/MnO_2_ com	Au	No	MnO_2_	No	Hydrothermal	1.4	0	1.4	2.6	25	[50]
Au/MnO_2_ NF	Au	No	MnO_2_ NF	No	Hydrothermal	1.3	0	1.3	2.6	60	[50]
Au/MnO_2_ NW	Au	No	MnO_2_ NW	No	Hydrothermal	1.3	0	1.3	3.2	45	[50]

^a^ Sol immobilization method using polyvinyl alcohol (PVA) as stabilizer and NaBH_4_ as a reductant.

**Table 5:** Catalysts used for the machine learning studies and the reaction conditions.

Catalyst	FFR/Metal Ratio [mol/mol]	Temp [°C]	Pressure [bar]	Reaction Time [h]	Solvent	pH	FA Yield [%]	Leaching **	Ref.
Au4Pd1@SiTi	50	110	26	10	Water	3	37	2	[62]
Au1Pd1@SiTi	50	110	26	10	Water	3	23	2	[62]
Au1Pd4@SiTi	50	110	26	10	Water	3	12	2	[62]
Au4Pd1/SiTi	50	110	26	10	Water	3	48	2	[62]
Au1Pd1/SiTi	50	110	26	10	Water	3	10	2	[62]
Au1Pd4/SiTi	50	110	26	10	Water	3	4	2	[62]
Au@SiTi	50	110	26	10	Water	3	10	2	[62]
Pd@SiTi	50	110	26	10	Water	3	7	2	[62]
HT-4:1	100	90	1	2	Water	7.6	1	1	[40]
Au/HT-4:1	100	90	1	2	Water	7.6	9	1	[40]
Au/HT-H_2_O_2_-4:1	100	90	1	2	Water + H_2_O_2_	7.6	19	1	[40]
Au/HT-4:1	100	90	1	6	Water	7.6	27	1	[40]
HT-2:1	100	90	1	2	Water	7	0	1	[40]
Au/HT-2:1	100	90	1	2	Water	7	6	1	[40]
Au/HT-H_2_O_2_-2:1	100	90	1	2	Water + H_2_O_2_	7	17	1	[40]
Au/HT-2:1	100	90	1	6	Water	7	20	1	[40]
HT-1:1	100	90	1	2	Water	6.5	0	1	[40]
Au/HT-1:1	100	90	1	2	Water	6.5	6	1	[40]
Au/HT-H_2_O_2_-1:1	100	90	1	2	Water + H_2_O_2_	6.5	22	1	[40]
Au/HT-1:1	100	90	1	6	Water	6.5	25	1	[40]
HT-1:5	100	90	1	2	Water	6	0	1	[40]
Au/HT-1:5	100	90	1	2	Water	6	0	1	[40]
Au/HT-H_2_O_2_-1:5	100	90	1	2	Water + H_2_O_2_	6	5	1	[40]
Au/HT-1:5	100	90	1	6	Water	6	3	1	[40]
Au/TiO_2_	50	110	15	2	Water	3	40.48	3	[65]
Pt/TiO_2_	50	110	15	2	Water	3	0.99	3	[65]
Pd/TiO_2_	50	110	15	2	Water	3	1.45	3	[65]
0.5%Pt1-Au1/TiO_2_	50	80	15	4	Water	3	21.8	3	[65]
1.25%Pt1-Au1/TiO_2_	50	80	15	4	Water	3	30.1	3	[65]
2%Pt1-Au1/TiO_2_	50	80	15	4	Water	3	24.9	3	[65]
0.5%Pt1-Au3/TiO_2_	50	80	15	4	Water	3	23.6	3	[65]
1.25%Pt1-Au3/TiO_2_	50	80	15	4	Water	3	32.7	3	[65]
2%Pt1-Au3/TiO_2_	50	80	15	4	Water	3	37.5	3	[65]
0.5%Pt3-Au1/TiO_2_	50	80	15	4	Water	3	15.2	3	[65]
1.25%Pt3-Au1/TiO_2_	50	80	15	4	Water	3	22.8	3	[65]
2%Pt3-Au1/TiO_2_	50	80	15	4	Water	3	15.6	3	[65]
0.5%Pd1-Au1/TiO_2_	50	80	15	4	Water	3	22.8	3	[65]
1.25%Pd1-Au1/TiO_2_	50	80	15	4	Water	3	29.9	3	[65]
2%Pd1-Au1/TiO_2_	50	80	15	4	Water	3	34.5	3	[65]
0.5%Pd1-Au3/TiO_2_	50	80	15	4	Water	3	19.8	3	[65]
1.25%Pd1-Au3/TiO_2_	50	80	15	4	Water	3	31.5	3	[65]
2%Pd1-Au3/TiO_2_	50	80	15	4	Water	3	34.1	3	[65]
0.5%Pd3-Au1/TiO_2_	50	80	15	4	Water	3	11.4	3	[65]
1.25%Pd3-Au1/TiO_2_	50	80	15	4	Water	3	14.8	3	[65]
2%Pd3-Au1/TiO_2_	50	80	15	4	Water	3	17.6	3	[65]
0.5%Pt1-Au1/TiO_2_	50	110	15	4	Water	3	8.8	3	[65]
1.25%Pt1-Au1/TiO_2_	50	110	15	4	Water	3	0	3	[65]
2%Pt1-Au1/TiO_2_	50	110	15	4	Water	3	0	3	[65]
0.5%Pt1-Au3/TiO_2_	50	110	15	4	Water	3	28.8	3	[65]
1.25%Pt1-Au3/TiO_2_	50	110	15	4	Water	3	0.9	3	[65]
2%Pt1-Au3/TiO_2_	50	110	15	4	Water	3	0	3	[65]
0.5%Pt3-Au1/TiO_2_	50	110	15	4	Water	3	5.2	3	[65]
1.25%Pt3-Au1/TiO_2_	50	110	15	4	Water	3	0	3	[65]
2%Pt3-Au1/TiO_2_	50	110	15	4	Water	3	0	3	[65]
0.5%Pd1-Au1/TiO_2_	50	110	15	4	Water	3	33.5	3	[65]
1.25%Pd1-Au1/TiO_2_	50	110	15	4	Water	3	24.5	3	[65]
2%Pd1-Au1/TiO_2_	50	110	15	4	Water	3	13.2	3	[65]
0.5%Pd1-Au3/TiO_2_	50	110	15	4	Water	3	51.6	3	[65]
1.25%Pd1-Au3/TiO_2_	50	110	15	4	Water	3	58.2	3	[65]
2%Pd1-Au3/TiO_2_	50	110	15	4	Water	3	58.7	3	[65]
0.5%Pd3-Au1/TiO_2_	50	110	15	4	Water	3	12.2	3	[65]
1.25%Pd3-Au1/TiO_2_	50	110	15	4	Water	3	0.9	3	[65]
2%Pd3-Au1/TiO_2_	50	110	15	4	Water	3	0	3	[65]
Au/MgO	50	110	26	2	Water	10.5	100	1	[41]
Au/TiO_2_	50	110	26	2	Water	3	30	1	[41]
Au/ZrO_2_	50	110	26	2	Water	4.9	32	1	[41]
Au/CeO_2_	50	110	26	2	Water	5	18	1	[41]
Au/MgF_2_	50	110	26	2	Water	3.8	2	1	[41]
Au/0.6MgF_2_-0.4MgO	50	110	26	2	Water	7.8	76	1	[41]
Au/0.4MgF_2_-0.6MgO	50	110	26	2	Water	8.8	99	1	[41]
SiO_2_@TiO_2_	100	110	24	2	Water	3	0.3	2	[64]
0.25% SiO_2_@Au@TiO_2_	100	110	24	2	Water	3	100	2	[64]
0.5% SiO_2_@Au@TiO_2_	100	110	24	2	Water	3	100	2	[64]
1% SiO_2_@Au@TiO_2_	100	110	24	2	Water	3	100	2	[64]
2% SiO_2_@Au@TiO_2_	100	110	24	2	Water	3	100	2	[64]
0.25% SiO_2_@TiO_2_@Au	100	110	24	2	Water	3	8.4	2	[64]
0.5% SiO_2_@TiO_2_@Au	100	110	24	2	Water	3	41.8	2	[64]
1% SiO_2_@TiO_2_@Au	100	110	24	2	Water	3	19	2	[64]
2% SiO_2_@TiO_2_@Au	100	110	24	2	Water	3	3.8	2	[64]
2%Au/CaO	50	110	15	2	Water	10	81	1	[66]
2%Au/SrO	50	110	15	2	Water	8.5	66	1	[66]
2%Au/BaO	50	110	15	2	Water	8	54	1	[66]
2%Au/BeO	50	110	15	2	Water	3.5	32	2	[66]
Au-HT2:1	200	110	6	2	Water	7	72	1	[38]
Au-HT3:1	200	110	6	2	Water	7	68	1	[38]
Au-HT4:1	200	110	6	2	Water	7	100	1	[38]
Au-HT5:1	200	110	6	2	Water	7	100	1	[38]
MnO_2_ com *	100	110	12	2	Water	5.5	2	3	[50]
MnO_2_ NF *	100	110	12	2	Water	5.5	14	3	[50]
MnO_2_ NW *	100	110	12	2	Water	5.5	4	3	[50]
Au/MnO_2_ com	100	110	12	2	Water	5.5	25	3	[50]
Au/MnO_2_ NF	100	110	12	2	Water	5.5	60	3	[50]
Au/MnO_2_ NW	100	110	12	2	Water	5.5	45	3	[50]

* FFR—support molar ratio, ** leaching expressed in 1 (high leaching), 2 (medium to low leaching), 3 (no leaching).

## Data Availability

The Data used for the machine learning optimization are available on request via corresponding author (R.W.).
